# Using Rapid Diagnostic Tests as a Source of Viral RNA for Dengue Serotyping by RT-PCR - A Novel Epidemiological Tool

**DOI:** 10.1371/journal.pntd.0004704

**Published:** 2016-05-09

**Authors:** Manivanh Vongsouvath, Koukeo Phommasone, Onanong Sengvilaipaseuth, Nathamon Kosoltanapiwat, Narisara Chantratita, Stuart D. Blacksell, Sue J. Lee, Xavier de Lamballerie, Mayfong Mayxay, Sommay Keomany, Paul N. Newton, Audrey Dubot-Pérès

**Affiliations:** 1 Department of Microbiology and Immunology, Faculty of Tropical Medicine, Mahidol University, Salaya, Thailand; 2 Lao-Oxford-Mahosot Hospital-Wellcome Trust Research Unit (LOMWRU), Microbiology Laboratory, Mahosot Hospital, Vientiane, Lao PDR; 3 Mahidol - Oxford Tropical Medicine Research Unit, Faculty of Tropical Medicine, Mahidol University, Salaya, Thailand; 4 Centre for Tropical Medicine and Global Health, Nuffield Department of Clinical Medicine, University of Oxford, Churchill Hospital, Oxford, United Kingdom; 5 UMR "Emergence des Pathologies Virales" (EPV: Aix-Marseille University - IRD 190 - Inserm 1207 - EHESP) & Fondation IHU Méditerranée Infection, APHM Public Hospitals of Marseille, Marseille, France; 6 Faculty of Postgraduate Studies, University of Health Sciences, Vientiane, Lao PDR; 7 Salavan Provincial Hospital, Salavan Provincial Health Department, Salavan Province, Laos; Hospital for Tropical Diseases, VIET NAM

## Abstract

**Background:**

Dengue virus infection causes major public health problems in tropical and subtropical areas. In many endemic areas, including the Lao PDR, inadequate access to laboratory facilities is a major obstacle to surveillance and study of dengue epidemiology. Filter paper is widely used for blood collection for subsequent laboratory testing for antibody and nucleic acid detection. For the first time, we demonstrate that dengue viral RNA can be extracted from dengue rapid diagnostic tests (RDT) and then submitted to real-time RT-PCR for serotyping.

**Methodology/Principal Findings:**

We evaluated the Standard Diagnostics (SD) Bioline Dengue Duo RDT, a commonly used test in dengue endemic areas. First, using the QIAamp RNA kit, dengue RNA was purified from the sample pad of the NS1 RDT loaded with virus isolates of the four serotypes, then quantified by RT-PCR. We observed greater recovery of virus, with a mean of 27 times more RNA recovered from RDT, than from filter paper. Second, we evaluated dengue NS1 RDTs from patients at Mahosot Hospital, Vientiane, (99 patients) and from rural Salavan Provincial Hospital (362 patients). There was good agreement between dengue RT-PCR from NS1 RDT with RT-PCR performed on RNA extracted from patient sera, either using RDT loaded with blood (82.8% and 91.4%, in Vientiane and Salavan, respectively) or serum (91.9% and 93.9%). There was 100% concordance between RDT and serum RT-PCR of infecting dengue serotype.

**Conclusions/Significance:**

Therefore, the collection of NS1 positive RDTs, which do not require cold storage, may be a novel approach for dengue serotyping by RT-PCR and offers promising prospects for the collection of epidemiological data from previously inaccessible tropical areas to aid surveillance and public health interventions.

## Author Summary

Dengue fever, caused by a virus transmitted by mosquitoes, is a public health problem in tropical and subtropical regions. Dengue Rapid Diagnostic Tests, in which a drop of blood is loaded onto a paper strip in a plastic cassette, are simple to use and have good diagnostic accuracy. They are becoming the test of choice for the management of dengue epidemics, especially in rural areas without laboratory facilities. However, four types of dengue virus circulate in most tropical areas and their patterns of circulation are of epidemiological importance since they play a role in the severity and propagation of the disease. We show, for the first time, that molecular amplification permitting dengue virus detection and typing can be performed directly from used positive RDTs, that can easily be transported. This novel approach has promising prospects for the collection of dengue epidemiological data from previously inaccessible tropical areas to aid surveillance and public health interventions.

## Introduction

The dengue virus (DENV) is an enveloped ssRNA flavivirus transmitted by *Aedes* mosquitoes [1]. Dengue infections are clinically classified by the World Health Organization (WHO) as dengue with or without warning signs and severe dengue [2]. It is an important public health problem affecting the tropical and subtropical world; Bhatt *et al*. estimate 390 million infections per year, of which 96 million present with clinical symptoms [3]. Approximately 2.4 billion people are currently at risk of dengue infection globally and most live in tropical and urban regions where the four dengue serotypes (DENV-1, 2, 3 and 4) circulate [4]. Secondary infections, which have been reported to be more severe than primary infections, occur when patients are sequentially infected with more than one serotype [5].

The combination of dengue NS1 antigen and anti-dengue IgM detection by ELISA is one of the standard diagnosis strategies, providing high sensitivity and high specificity, covering the viremic phase at the early course of the disease and a later phase when viral RNA is no longer detectable in blood, respectively [6,7]. Gene segment amplification by reverse transcription followed by polymerase chain reaction (RT-PCR) is widely applied for the detection of dengue virus during the viraemic phase, with the advantage of permitting dengue serotyping.

In Lao PDR (Laos), dengue infection is a major cause of morbidity with a rising case fatality rate [8]. Approximately 3.9 million residents are presently at risk of dengue infection [9]. It is usually regarded as an urban disease but recent studies in Laos suggest that it is also an important rural disease [10–12]. However, only limited data are available on dengue epidemiology in Laos as only few institutions, located in the capital city (Vientiane), have access to laboratory facilities required to perform ELISA and RT-PCR and the transportation of frozen specimens is very difficult.

Immunochromatographic Rapid Diagnostic Tests (RDTs), of which a variety of different brands are available for diagnosing dengue, are alternatives for diagnosis in rural areas. They are rapid, accurate, easy to use and do not require advanced technical knowledge or equipment. The Standard Diagnostics (SD) Bioline Dengue Duo RDT (SD dengue RDT; Standard Diagnostics, Kyonggi-do, Korea) permits the concomitant detection of dengue NS1 antigen and anti-dengue IgM and IgG antibodies with overall sensitivity and specificity greater than 80% [13,14]. This dengue RDT remained stable at elevated temperature over 2 years storage in Laos [15]. Dengue RDTs are now used in provincial hospitals and in a few health centers in southern Laos, and are likely to be extended in rural areas.

However, such RDTs do not give information on the infecting serotype, important for both public health surveillance and dengue epidemiology research. We therefore hypothesized that dengue virus could be extracted from NS1 positive RDTs for serotype determination and that such a system could be used for dengue serotype surveillance by the sending of positive RDTs to a central laboratory for RT-PCR. RNA detection from dried blood spot (DBS) for measles, HIV-1, Hepatitis C, dengue and Chikungunya viruses have been described [16–21], but detection of pathogen nucleic acid by PCR from RDTs has only been described for *Salmonella enterica* serovar Typhi and *Plasmodium falciparum* [22,23].

We therefore compared techniques for dengue RNA extractions for the four dengue serotypes, followed by RT-PCR, from SD dengue RDTs, filter papers and neat samples. Evaluation was then performed in two clinical cohorts in Laos, in a central and a rural hospital, of patients with suspected dengue.

## Methods

### Description of RDT

The SD dengue RDT is an in vitro immunochromatographic assay for the detection of dengue virus NS1 Ag and anti-dengue IgM/IgG antibodies in human serum, plasma, or whole blood, from finger-prick or venous blood. This test comprises a pair of test devices, a dengue NS1 Ag test on the left side, and a dengue IgM/IgG antibody (Ab) test on the right side. Each device contains a strip, enclosed in a plastic cassette. The strip is made of three compartments; i) an absorptive pad where the patient sample (serum, blood or plasma) is applied and then moves along the strip, ii) a conjugate or reagent pad which contains antibodies specific to the target analytic conjugated to colored particles, iii) a nitrocellulose membrane on which the immunocomplexes move until the zone of reaction where they are immobilized and appear as a colored band. The test is easy to perform—three drops (using dropper provided with the kit, ~100 μL) and 10 μL (using a capillary provided with the kit) of sample are applied into the two small wells on the NS1 and Ab cassettes, respectively. Four drops of diluent (provided with the kit) are then applied on the Ab cassette. The test results are obtained in 15 minutes.

### Sample collection

Samples were collected at two sites: Mahosot Hospital, a central hospital in Vientiane Capital, and Salavan Provincial Hospital, in a rural area of southern Laos 679 km to the south-east (15.72 N, 106.42 E).

At Mahosot Hospital, 99 consenting patients admitted with symptoms meeting the WHO criteria [2] for dengue infection were enrolled from August to November 2013. At Salavan hospital, 362 consenting patients with undifferentiated fever who tested negative by malaria RDT (SD Bioline Malaria Ag P.f/P.v) were enrolled from July to October 2012. Patient information is displayed in supporting information (S1 Table).

Venous blood alone was collected from patients at Mahosot Hospital. SD dengue RDTs were performed according to manufacturer’s recommendations using whole blood and, after whole blood centrifugation, serum. The dropper provided with the RDT and a micropipette set at 100μl were used to load whole blood and serum, respectively, on NS1 cassettes. Two drops of whole blood and one hundred microliters of serum were loaded on filter paper (FP, Grade 0903 Whatman, GE Healthcare) and 0.2 ml of serum was kept at -80°C as a reference neat serum sample.

At Salavan Hospital, both venous whole blood and capillary whole blood from finger pricks were collected. SD dengue RDTs were performed according to manufacturer’s recommendations using capillary whole blood and, after capillary whole blood centrifugation, serum. Whole blood was directly dropped onto the NS1 cassette and a micropipette was used to load 100μl of serum. Two drops of whole capillary blood were loaded on filter paper. After venous blood centrifugation, 0.2 ml of serum was kept at -20°C as a reference neat serum sample for each patient.

FP and RDT were dried at room temperature for 2 hours, put in individual plastic zip lock bags with desiccant and stored, at room temperature in Salavan and directly into -80°C at Mahosot Hospital until analysis. Samples were shipped from Salavan to the Microbiology Laboratory, Mahosot Hospital, on dry ice for serum and in metals boxes at ambient temperature for FP and RDT once a month. All specimens arriving at Mahosot Hospital were immediately kept at -80°C until testing.

### Ethics statement

Written informed consent was obtained from all recruited patients or responsible guardians. The patients were recruited in the framework of two studies with ethical approval by the Lao National Ethics Committee for Health Research and the Oxford Tropical Research Ethics Committee (OXTREC).

### Virus isolates preparation from virus-infected Vero cells

Sera from dengue patients, infected with one of all four dengue serotypes, admitted at Mahosot Hospital (diagnosed and serotyped by serum RT-PCR) [24] were inoculated on Vero cells as described [10]. After seven days of incubation at 37°C in 5% CO_2_, virus isolates (DENV-1, 2, 3, and 4) were recovered from the supernatant after centrifugation of cell culture medium. Ten fold serial dilutions using minimum essential medium (MEM, Gibco) were performed for each isolate and aliquots were stored at -80°C for subsequent experiments. One hundred microliters of each dilution were loaded on RDT NS1 cassette and FP, then stored as described above.

### RDT and filter paper processing

**Fig 1:**
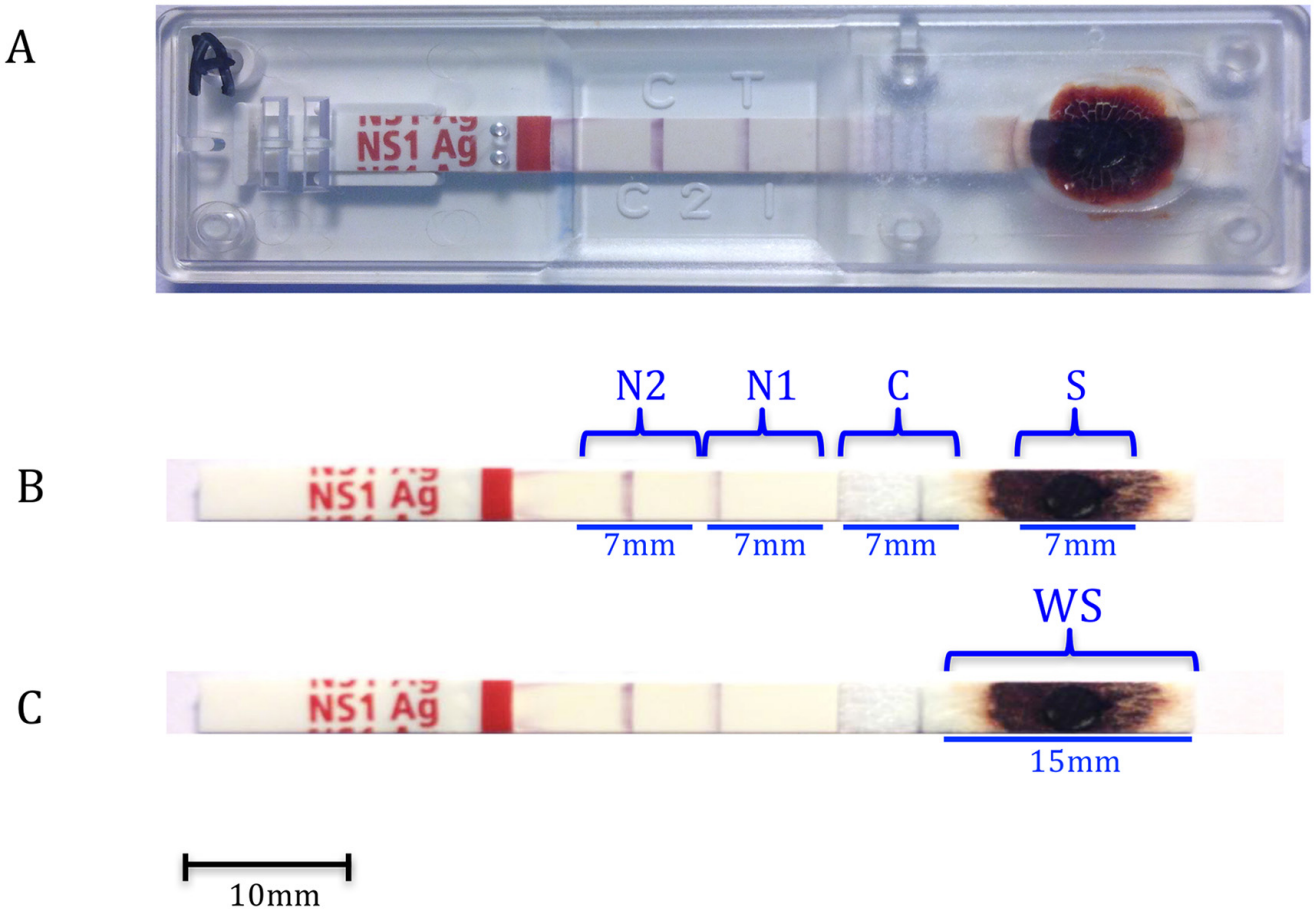
NS1 RDT strip cutting. A: NS1 cassette. B: strip outside the cassette with the location of the 4 parts cut for the experiment, N: nitrocellulose, C: conjugate pad, S: sample pad, N1 and N2: nitrocellulose membrane. C: strip with the location of the whole S pad (WS) cut for the experiment.

RDT strips and FPs, loaded with samples, were cut just before RNA extraction. The RDT NS1 cassette was opened using forceps and the strip taken out. The strip was cut, with a sterile scalpel, into four sections of 7 mm length each, from sample pad (S), conjugate pad (C) and nitrocellulose membrane (two pieces, N1 and N2) (see Fig 1). Subsequently, to improve the quantity of dengue RNA recovered from RDTs, additional experiments were performed by cutting out the whole sample pad (WS), obtaining a section of 15 mm length (Fig 1). Two discs of 6 mm diameter were punched from FP at the middle of the sample spot, using a single hole puncher.

### RNA extraction

140 μl of each sample of virus isolates and sera were extracted using QIAamp Viral RNA Minikit (QIAGEN AG, Hombrechtikon, Switzerland), following manufacturer instructions (elution in 60μl).

RDT sections and FP discs were processed according to the procedure described for dried swabs in the EZ1 Virus Mini Kit v2.0 handbook (Qiagen). They were incubated for 15 minutes at 56°C with 200μl of ATL lysis buffer (Qiagen) and then 140 μl of the mixture was extracted using the QIAamp Viral RNA Minikit (Qiagen), following manufacturer’s instructions, with 60μl elution volume.

### Dengue RT-PCR

For the detection of dengue RNA after extraction, the pan-dengue Taqman real-time RT-PCR system (DENV All RT-PCR) developed by Leparc-Goffard *et al*. [24] was used with four serotype-specific RT-PCRs. The SuperScript III Platinum One-Step qRT-PCR kit (Invitrogen) with 200nM of each primer and 100nM of probe on 5μl of RNA extract was used. Synthetic RNA control was prepared as described by Ninove *et al*. [25]. Three serial dilutions, 2.5 x10^6^, 2.5 x10^4^ and 2.5 x10^2^ copies/μl of positive control were prepared and aliquoted at -80°C and used as standards in each RT-PCR run. All samples and standards were tested by DENV All RT-PCR in duplicate. Means of Ct values of the duplicates were used for the quantification of dengue RNA copies in tested samples (supporting information, S2 Table).

### Assessment of the extraction techniques using virus isolates

For each of the four serotypes, three virus isolate dilutions (4.3 x10^4^, 4.3 x10^5^ and 4.3 x10^6^ copies/ml) were used to assess the extraction technique. Each of the three dilutions, for the four serotypes, was loaded in triplicate on RDTs and on FP (supporting information, S1 Fig). All RDT and FP samples underwent separate extraction along with 140μl of the 12 virus dilutions as comparators. All extracts underwent DENV All RT-PCR for RNA quantification. The mean number of dengue RNA copies was calculated from triplicate extractions, with the relative standard deviation (RSD), to assess the reproducibility of the technique. Dengue RNA extractions from RDTs and FPs were compared to direct extraction by dividing the number of copies obtained from RDTs and FPs by the number of copies obtained by the direct extraction of virus solution. This was expressed as a percentage of RNA recovery (multiplying the ratio by 100); 100% indicating that the number of RNA copies obtained after RDT or FP extraction was the same as from direct extraction.

### Statistical analysis

The techniques developed in this study for RNA preparation from RDT and filter paper were compared to the direct RNA extraction from neat serum. In the absence of gold standard, outcomes (dengue RT-PCR results) are presented in a 2x2 table and agreements (95% confidence intervals) were calculated, as recommended by US FDA [26], using Stata v10 [27]. The agreements of the different RNA preparation techniques were then compared using the z test.

## Results

### Assessment of extraction techniques using virus isolates

**Fig 2:**
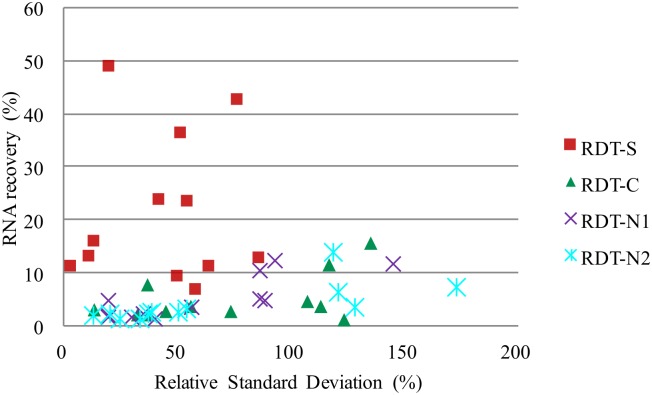
Efficiency of dengue RNA extraction from different RDT parts (S, C, N1 and N2) compared to the direct extraction for all isolate dilutions for the four dengue serotypes. In y axis: RNA recovery in percentage: (RDT/Dir)*100 = ratio of the mean number of dengue RNA copies/μl recovered by RDT extraction (S, C, N1 or N2 parts) over the mean number of dengue RNA copies/μl recovered by the direct extraction. X-axis: RSD = relative standard deviation for the different extraction techniques.

Dengue RNA was detected after extraction from all the 4 sections of the NS1 RDT strip, even for the isolates with the lowest dengue copy concentration (4.3 x10^4^ copies/ml). The recovery of dengue RNA from RDTs was less efficient than the direct extraction of the virus isolate (Table 1). RDT-S extraction permitted recovery of 7 to 49% of the quantity of RNA recovered by direct extraction and was much more efficient than the extraction from the other RDT sections (Fig 2). The C, N1 and N2 RDT sections permitted recovery of 1–16%, 1–12% and 1–14%, respectively, of the quantity of RNA recovered by direct extraction. Extraction from the S section permitted the best reproducibility with the lowest RSD from 3 to 86% whereas the RSD for C, N1 and N2 sections were 13–135%, 19–145% and 13–173%, respectively.

**Table 1:** Mean number of dengue RNA copies/μl recovered by extraction from the 4 parts of RDT strip compared to the direct extraction.

Virus isolate dilutions	DENV-1			DENV-2			DENV-3			DENV-4		
	10^4^	10^5^	10^6^	10^4^	10^5^	10^6^	10^4^	10^5^	10^6^	10^4^	10^5^	10^6^
Direct extraction												
Mean co/μl	450	4 500	58 000	310	3 100	16 000	590	5 100	48 000	280	2 300	16 000
RSD (%)	4	8	9	34	36	9	13	10	17	32	16	30
(Dir/Dir)*100	100	100	100	100	100	100	100	100	100	100	100	100
Extraction from RDT S part												
Mean co/μl	41	1 100	7 600	110	1 500	6 900	39	570	5 500	36	540	2 600
RSD (%)	50	54	11	51	20	77	58	64	3	86	42	13
(S/Dir)*100	9	23	13	36	49	42	7	11	11	13	24	16
Extraction from RDT C part												
Mean co/μl	70	170	1 200	14	79	1 300	12	160	5 500	3	65	610
RSD (%)	135	56	38	107	45	37	33	13	117	123	73	113
(C/Dir)*100	16	4	2	4	3	8	2	3	11	1	3	4
Extraction from RDT N1 part												
Mean co/μl	21	210	6 000	16	110	2 000	8	95	5 600	4	39	350
RSD (%)	20	89	87	87	56	93	40	20	145	19	30	35
(N1/Dir)*100	5	5	10	5	4	12	1	2	12	1	2	2
Extraction from RDT N2 part												
Mean co/μl	11	120	1 300	23	93	2 200	21	110	3 000	4	28	300
RSD (%)	51	38	37	173	54	119	128	20	121	33	25	13
(N2/Dir)*100	2	3	2	7	3	14	4	2	6	1	1	2

Mean co/μl = mean of DENV RNA copies/μl recovered after each extraction, calculated from number of copies obtained in the triplicated extractions (displayed in supporting information). S or C or N1 or N2/Dir = ratio of the mean number of dengue RNA copies recovered after RDT extraction over the mean number of dengue RNA copies recovered by direct extraction. Virus isolate dilution 10^4^ = dilutions with a virus titer of 4.3x10^4^ copies/ml, 10^5^ = 4.3x10^5^ copies/ml and 10^6^ = 4.3x10^6^ copies/ml.

To improve dengue RNA extraction from NS1 RDTs, the Whole S pad (WS, 15mm) was tested. The quantities of DENV RNA copies recovered from RDT-WS were higher than from RDT-S for all 4 DENV serotypes for all DENV isolate dilutions. The extractions from RDT-WS permitted recovery of 34%-169% of the quantity of RNA recovered by the direct extraction, in contrast to 7% to 49% for RDT-S extraction (Table 2). Therefore, extraction from RDT-WS was selected for subsequent experiment using patient samples.

**Table 2:** Mean number of dengue RNA copies/μl recovered by extraction from RDT S, WS parts and filter paper, compared to direct extraction.

Virus isolate dilutions	DENV-1			DENV-2			DENV-3			DENV-4		
	10^4^	10^5^	10^6^	10^4^	10^5^	10^6^	10^4^	10^5^	10^6^	10^4^	10^5^	10^6^
Direct extraction												
Mean co/μl	450	4 500	58 000	310	3 100	16 000	590	5 100	48 000	280	2 300	16 000
RSD (%)	4	8	9	34	36	9	13	10	17	32	16	30
(Dir/Dir)*100	100	100	100	100	100	100	100	100	100	100	100	100
Extraction from RDT S part												
Mean co/μl	41	1 100	7 600	110	1 500	6 900	39	570	5 500	36	540	2 600
RSD (%)	50	54	11	51	20	77	58	64	3	86	42	13
(S/Dir)*100	9	23	13	36	49	42	7	11	11	13	24	16
Extraction from RDT WS part												
Mean co/μl	420	4 800	33 000	310	2 900	28 000	300	1 700	24 000	130	1 400	13 000
RSD (%)	76	76	73	81	73	77	79	80	78	79	73	76
(WS/Dir)*100	94	106	58	100	93	169	50	34	49	45	60	80
Extraction from FP												
Mean co/μl	21	160	2 700	5	62	720	34	220	2 300	5	43	410
RSD (%)	5	17	13	44	17	20	33	32	19	37	12	23
(FP/Dir)*100	5	4	5	2	2	4	6	4	5	2	2	3

Mean co/μl = mean of DENV RNA copies/μl recovered after each extraction, calculated from number of copies obtained in the triplicated extractions (displayed in supporting information). S, WS or FP /Dir = ratio of the mean number of dengue RNA copies recovered after RDT of filter paper extraction over the mean number of dengue RNA copies recovered by direct extraction. Virus isolate dilution 10^4^ = dilutions with a virus titer of 4.3x10^4^ copies/ml, 10^5^ = 4.3x10^5^ copies/ml and 10^6^ = 4.3x10^6^ copies/ml.

**Fig 3:**
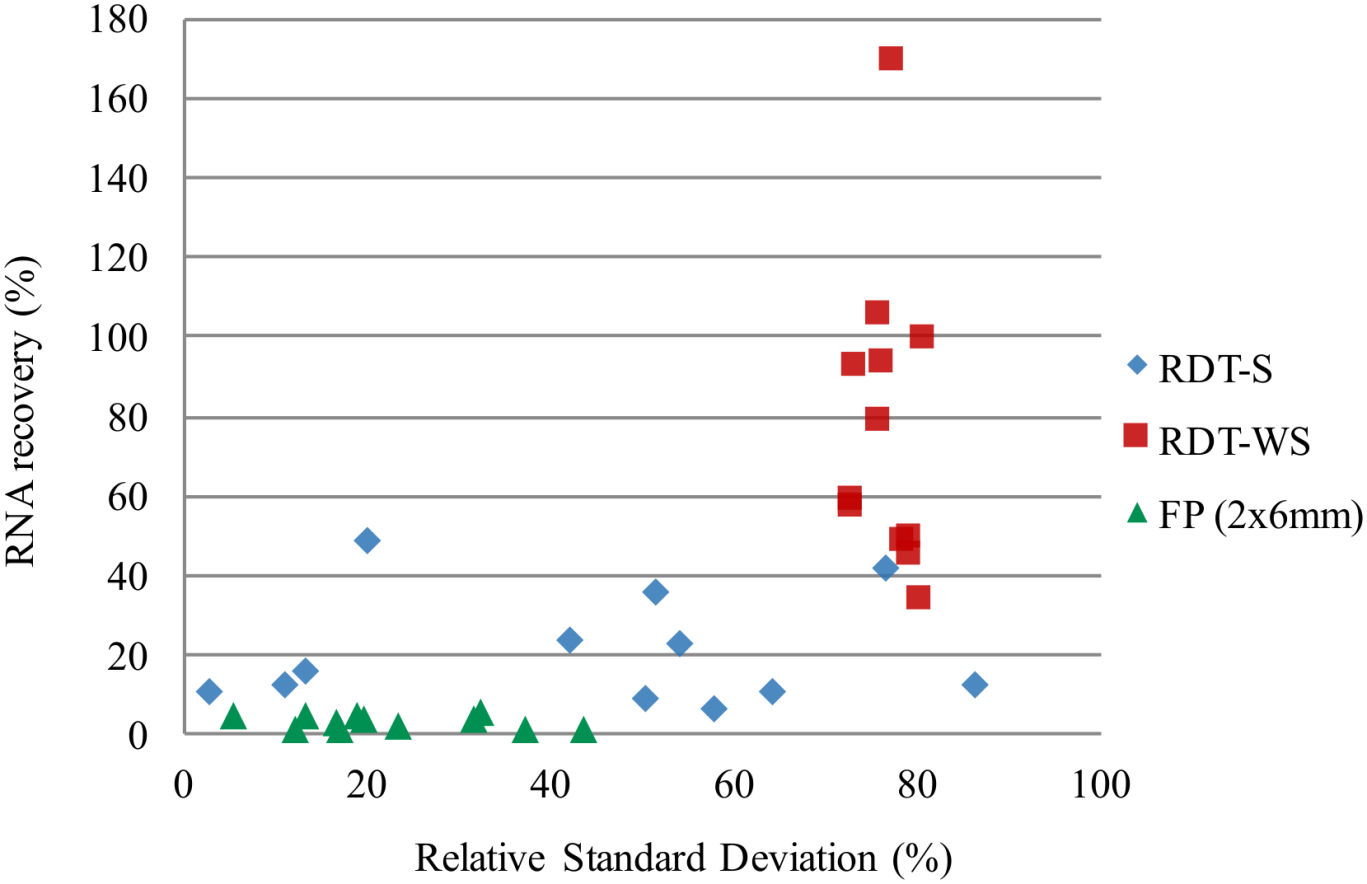
Efficiency of dengue RNA extraction from RDT-S part (7mm), RDT-WS part (15 mm) and FP (2 disc of 6mm) compared to the direct extraction for all isolate dilutions for the four dengue serotypes. In y axis: RNA recovery in percentage: (RDTor FP/Dir)*100 = ratio of the mean number of dengue RNA copies/μl recovered by RDT(S part or WS: whole S part) or FP extraction over the mean number of dengue RNA copies/μl recovered by the direct extraction. X axis: RSD = relative standard deviation for the different extraction techniques.

The RNA recovery from FP was much less efficient with only 2 to 6% of the quantity recovered by the direct extraction (Table 2, Fig 3). On average, 27 times less dengue RNA copies were recovered from FP than from RDT-WS.

### Evaluation using patient samples

#### Central hospital

For the 99 patients recruited at Mahosot Hospital, RNA extractions from NS1 RDTs and FP were compared to neat serum extraction based on DENV All RT-PCR results (Table 3). Percent agreements with neat serum extraction (overall, positive and negative) were calculated (95% CI) for all techniques (Table 3). The overall, positive and negative percent agreements for NS1 RDTs with serum and whole blood added were, respectively, for serum: 91.9% (84.7–96.4%), 85.4% (72.2–93.9%) and 98.0% (89.6–99.9%); for whole blood: 82.8% (73.9–89.7%), 70.8% (55.9–83.0%) and 94.1% (83.8–98.8%).

**Table 3:** Number of patients found positive by DENV. All RT-PCR performed after extraction from RDT or filter paper and from neat serum for 99 patients admitted at Mahosot Hospital.

A	Serum on RDT	Neat serum			
		Positive		Negative	Total
	Positive	41		1	42
	Negative	7		50	57
	Total	48		51	99
	Overall agreement (95%CI): 91.9 (84.7–96.4)%				
	Positive agreement (95%CI): 85.4 (72.2–93.9)%				
	Negative agreement (95%CI): 98.0 (89.6–99.9)%				
B	Whole blood on RDT	Neat serum			
		Positive		Negative	Total
	Positive	34		3	37
	Negative	14		48	62
	Total	48		51	99
	Overall agreement (95%CI): 82.8 (73.9–89.7)%				
	Positive agreement (95%CI): 70.8 (55.9–83.0)%				
	Negative agreement (95%CI): 94.1 (83.8–98.8)%				
C	Serum on filter paper		Neat serum		
			Positive	Negative	Total
	Positive		34	0	34
	Negative		14	51	65
	Total		48	51	99
	Overall agreement (95%CI): 85.9 (77.4–92.0)%				
	Positive agreement (95%CI): 70.8 (55.9–83.0)%				
	Negative agreement (95%CI): 100 (93.0–100)%				
D	Whole blood on filter paper	Neat serum			
		Positive		Negative	Total
	Positive	35		0	35
	Negative	13		51	64
	Total	48		51	99
	Overall agreement (95%CI): 86.9 (78.6–92.8)%				
	Positive agreement (95%CI): 72.9 (58.2–84.7)%				
	Negative agreement (95%CI): 100 (93.0–100)%				

A: Serum on RDT compared to neat serum extraction. B: Whole blood on RDT compared to neat serum extraction. C: serum on filter paper compared to neat serum extraction. D: Whole blood on filter paper compared to neat serum extraction.

There were no significant differences in agreements (all p>0.05) between neat serum extraction and all the RNA preparation methods (Table 4). The highest positive agreement with the neat serum extraction was with serum on RDTs. Negative agreement with the neat serum extraction was high for all techniques investigated and best with the whole blood filter paper.

**Table 4:** Comparison of agreements of the different techniques for RNA preparation with the neat serum extraction for Mahosot Hospital samples.

RNA preparation tested	% Agreement with neat serum (p value)								
	Overall			Positive			Negative		
S RDT / WB RDT	91.9	82.8	(0.054)	85.4	70.8	(0.084)	98.0	94.1	(0.312)
S FP / WB FP	85.9	86.9	(0.837)	70.8	72.9	(0.819)	100	100	-
S RDT / S FP	91.9	85.9	(0.179)	85.4	70.8	(0.084)	98.0	100	(0.310)
WB RDT / WB FP	82.8	86.9	(0.421)	70.8	72.9	(0.819)	94.1	100	(0.078)

S RDT: extraction from serum on RDT, S FP: extraction from serum on filter paper, WB RDT: extraction from whole blood on RDT, WB FP: extraction from whole blood on filter paper.

To assess if differences were observed according to RDT results and duration of illness, the same agreements comparison were performed for three group of patients: those NS1 positive, those presenting with less than 5 days of illness and those presenting with 5 or more days of illness (S3 Table). No significant differences were observed.

Only one and three patients from Mahosot Hospital were PCR positive after extraction from serum on RDT and whole blood on RDT, respectively, and negative after neat serum extraction.

All dengue positive neat, RDT and FP samples were tested by real-time RT-PCR for serotyping (S4 Table). All four DENV serotypes were found, with the majority of patients having DENV-3 (81%; 42/52) followed by DENV-2 (10%; 5/52), DENV-4 (4%; 2/52), and DENV-1 (4%; 2/52) with one sample that could not be typed. There was 100% concordance between RDT and serum RT-PCR of infecting dengue serotype.

#### Rural hospital

For the 362 patients recruited at Salavan, RNA extractions from NS1 RDTs and FP were compared to neat serum extraction based on DENV All RT-PCR results (Table 5). Percent agreements with neat serum extraction (overall, positive and negative) were calculated (95% CI) for all techniques (Table 5). The overall, positive and negative percent agreements for NS1 RDTs were, respectively, for serum: 93.96% (90.96–965.29%), 943.01% (876.42–97.82%), and 93.9% (90.32–96.5%); and for whole blood: 910.46% (887.1–943.14%), 920.01% (842.85–965.51%) and 910.28% (876.16–94.40%). There were no significant differences in agreements (all p>0.05) between neat serum extraction using RDT or filter paper, loaded with either blood or serum (Table 6).

**Table 5:** Number of patients positive by DENV. All RT-PCR performed after extraction from RDT or filter paper and from neat serum for 362 patients admitted at Salavan Provincial Hospital.

A	Serum on RDT	Neat serum		
		Positive	Negative	Total
	Positive	94	16	110
	Negative	6	246	252
	Total	100	262	362
	Overall agreement (95%CI): 93.9 (90.9–96.2)%			
	Positive agreement (95%CI): 94.0 (87.4–97.8)%			
	Negative agreement (95%CI): 93.9 (90.3–96.5)%			
B	Whole blood on RDT	Neat serum		
		Positive	Negative	Total
	Positive	92	23	115
	Negative	8	239	247
	Total	100	262	362
	Overall agreement (95%CI): 91.4 (88.1–94.1)%			
	Positive agreement (95%CI): 92.0 (84.8–96.5)%			
	Negative agreement (95%CI): 91.2 (87.1–94.4)%			
C	Whole blood on filter paper	Neat serum		
		Positive	Negative	Total
	Positive	89	24	113
	Negative	11	238	249
	Total	100	262	362
	Overall agreement (95%CI): 90.3 (86.8–93.2)%			
	Positive agreement (95%CI): 89.0 (81.2–94.4)%			
	Negative agreement (95%CI): 90.8 (86.7–94.0)%			

A: Serum on RDT compared to neat serum extraction. B: Whole blood on RDT compared to neat serum extraction. C: Whole blood on filter paper compared to neat serum extraction.

**Table 6:** Comparison of agreements of the different techniques for RNA preparation with the neat serum extraction for Salavan Provincial Hospital sample.

RNA preparation tested	% Agreement with neat serum (p value)								
	Overall			Positive			Negative		
S RDT / WB RDT	93.9	91.4	(0.197)	94.0	92.0	(0.579)	93.9	91.2	(0.239)
WB RDT / WB FP	91.4	90.3	(0.608)	92.0	89.0	(0.469)	91.2	90.8	(0.873)
S RDT / WB FP	93.9	90.3	(0.073)	94.0	89.0	(0.205)	93.9	90.8	(0.182)

S RDT: extraction from serum on RDT, WB RDT: extraction from whole blood on RDT, WB FP: extraction from whole blood on filter paper.

For NS1 positive patients, the overall agreement with neat serum extraction was significantly higher (90%) for serum on RDT than for whole blood on filter paper (80.2%), p value 0.040 (S5 Table).

Some patients’ samples were negative by PCR after neat serum extraction but positive after extraction from serum on RDT (16 patients), whole blood on RDT (23) or whole blood on FP (24).

The dengue serotypes at Salavan were mostly DENV-1 (80%; 113/142) followed by DENV-2 (12%; 17/142) and DENV-3 (4%; 6/142). No patients with DENV-4 serotype were detected and samples from six patients could not be typed. There was 100% concordance between RDT and serum RT-PCR of infecting dengue serotype.

## Discussion

These results suggest that dengue serotype can be determined by PCR of the NS1 pad of one brand of dengue RDT, which is an potentially useful tool for the large populations without access to laboratory facilities.

Prado *et al*. [17] and Matheus *et al*. [20] reported detection of dengue virus by RT-PCR from dried blood spots on filter paper. The former study tested dengue 2 and dengue 3 viruses from FP from 52 patients and the latter tested FP from 666 NS1 positive patients. Here we found similar results with good overall agreement between neat serum and FP extraction either loaded with serum or whole blood from patients at Mahosot Hospital. On evaluation under field conditions, 362 patient samples collected on filter paper from Salavan, stored for 1 month at room temperature (18 to 46°C) [15], also showed good overall agreement of 90.3% between filter paper extraction and neat serum extraction. The extraction technique used has the advantage of being a simple commercial kit, without phenol. However, the use of Trizol remains a good alternative for laboratories with limited resources.

Although, *P*. *falciparum* and *S*. Typhi DNA detection by PCR has been achieved from RDTs, [22,23,28,29] to the best of our knowledge virus detection by RT-PCR from RDTs has not been described. Studies on *Plasmodium* DNA detection from RDTs showed that different components of the strip demonstrated variable suitability for nucleic acid purification. Cnops *et al*. [29] found the nitrocellulose membrane to be the most suitable area whereas Veron and Carne [28] and Ishengoma *et al*. [22] found that it was the sample pad. For SD dengue RDT our data show that the sample pad area from NS1 cassette is the best section for dengue RNA detection. The efficiency in RNA recovery from the full WS part (15mm) was close to what was obtained by direct neat sample extraction (34% to 169% RNA recovery). This shows the importance of assessing the optimal RDT section to be used for PCR. Whether this varies between dengue NS1 RDT brands remains to be determined. Interestingly, the RNA recovery from RDTs was 27 times more efficient than from 2 discs (6mm) of filter paper. The evaluation on patient samples from a central and a provincial hospital showed good overall agreements between neat sera and RDT extraction for all conditions (82.8% to 93.9%) with no significant differences when using RDT or filter paper, loaded with blood or serum, for the detection of DENV by RT-PCR (all comparison of agreements p>0.05). However, for NS1 positive patients from Salavan, better agreement was observed for RDT in comparison to filter paper. Some patients were found negative by PCR from neat serum and positive from RDT or filter paper, 42 patients from Salavan and only 4 from Mahosot. This difference is probably due to the suboptimal storage at -20°C of sera in Salavan. In addition, RDTs and filter papers from individual patients were kept in individual zip lock bag to avoid contamination but we can not exclude that this process was not strictly followed and that contamination between bloody RDTs and filter papers could have happened.

Although the patient populations we tested were infected by all four serotypes, patients from Mahosot Hospital were mainly infected by DENV-3 (83.3%), reflecting the DENV-3 outbreak occurring at that time in Vientiane [30] and those from Salavan mainly by DENV-1 (88.9%). Therefore, it was not possible to evaluate potential differences in RDT and FP dengue detection according to serotype. Additional studies are needed to better assess the effect of temperature storage in the efficiency of RNA recovery from RDT. Moreover, this study was performed in Salavan Provincial Hospital where staff are familiar with sample collection for testing in the central hospital. Study at other provincial and district hospitals, and eventually health centers, over a longer period would be useful to assess the sustainability of this strategy. And finally it would be important to test this process using other dengue RDT brands to see if this technique could be generalized.

Dengue RDTs are becoming important diagnosis tools in dengue epidemic management and is the only diagnostic test, when any are available, in provincial hospitals and health centers in Laos. It is expected that their use will be extended into more remote areas [15]. Therefore, positive dengue RDTs could be, in Laos and elsewhere in rural Asia, appropriate devices for sample storage and easy transportation to higher-level facilities for dengue serotype RT-PCR determination. RDTs and dried blood spots (DBS) should be considered as potentially infectious and thus handled appropriately [31]. One might imagine that the collection of used RDT could become a standard procedure, however this would require additional experiments to assess if RDT could be modified, as by pre or post chemical treatment, to improve RNA preservation and recovery. Although filter paper is of low cost and easily distributed it would not be needed where dengue RDTs are used for routine diagnosis. Used NS1 RDTs could be collected and transported for batched RT-PCR by national surveillance programs.

This technique may also permit dengue envelope sequencing for deeper molecular epidemiology analysis from RNA purified from RDTs. This could greatly increase availability of dengue epidemiological data from previously inaccessible tropical areas by facilitating dengue confirmation tests and strain identification to aid surveillance and public health interventions. This will also be of considerable importance if dengue vaccines are introduced. In addition, negative dengue RDTs could also be evaluated for PCR for other viruses causing similar clinical syndromes, such as chikungunya and zika viruses, to aid in differential diagnosis. As RDTs become increasingly used for a diversity of diseases, further exploration to look at what ‘added value’ could be extracted could be important for public health.

## Supporting Information

S1 Fig Overview of all extractions performed with each virus isolate dilution. (TIF)

S1 Table Clinical feature and laboratory RDT results for patients included in the study. (DOCX)

S2 Table Quantification by DENV All RT-PCR of dengue RNA recovered by FP, RDT and direct extractions from virus isolates. Dir Ext: Direct extraction, FP: filter paper. Virus isolate dilution 104 = dilutions with a virus titer of 4.3x104 copies/ml, 105 = 4.3x105 copies/ml and 106 = 4.3x106 copies/ml.
(DOCX)

S3 Table Agreements in RT-PCR results with neat serum extraction for all RNA purification techniques for groups of patients from Mahosot. A: patients NS1 positive by RDT. B: patients with less than 5 days of fever on admission. C: patients with more than 5 days of fever on admission.
(XLSX)

S4 Table Results of serotyping RT-PCR performed for each RNA purification. A: For the 99 patients admitted at Mahosot Hospital. B: For the 362 patients admitted at Salavan Hospital.
(DOCX)

S5 Table Agreements in RT-PCR results with neat serum extraction for all RNA purification techniques for groups of patients from Salavan. A: patients NS1 positive by RDT. B: patients with less than 5 days of fever on admission. C: patients with more than 5 days of fever on admission.
(XLSX)
