# Supplementary figures and images for: Using Rapid Diagnostic Tests as a Source of Viral RNA for Dengue Serotyping by RT-PCR - A Novel Epidemiological Tool
Source: PLoS Negl Trop Dis. 2016 May 9;10(5):e0004704. doi: 10.1371/journal.pntd.0004704 (PMC4861341; doi:10.1371/journal.pntd.0004704)

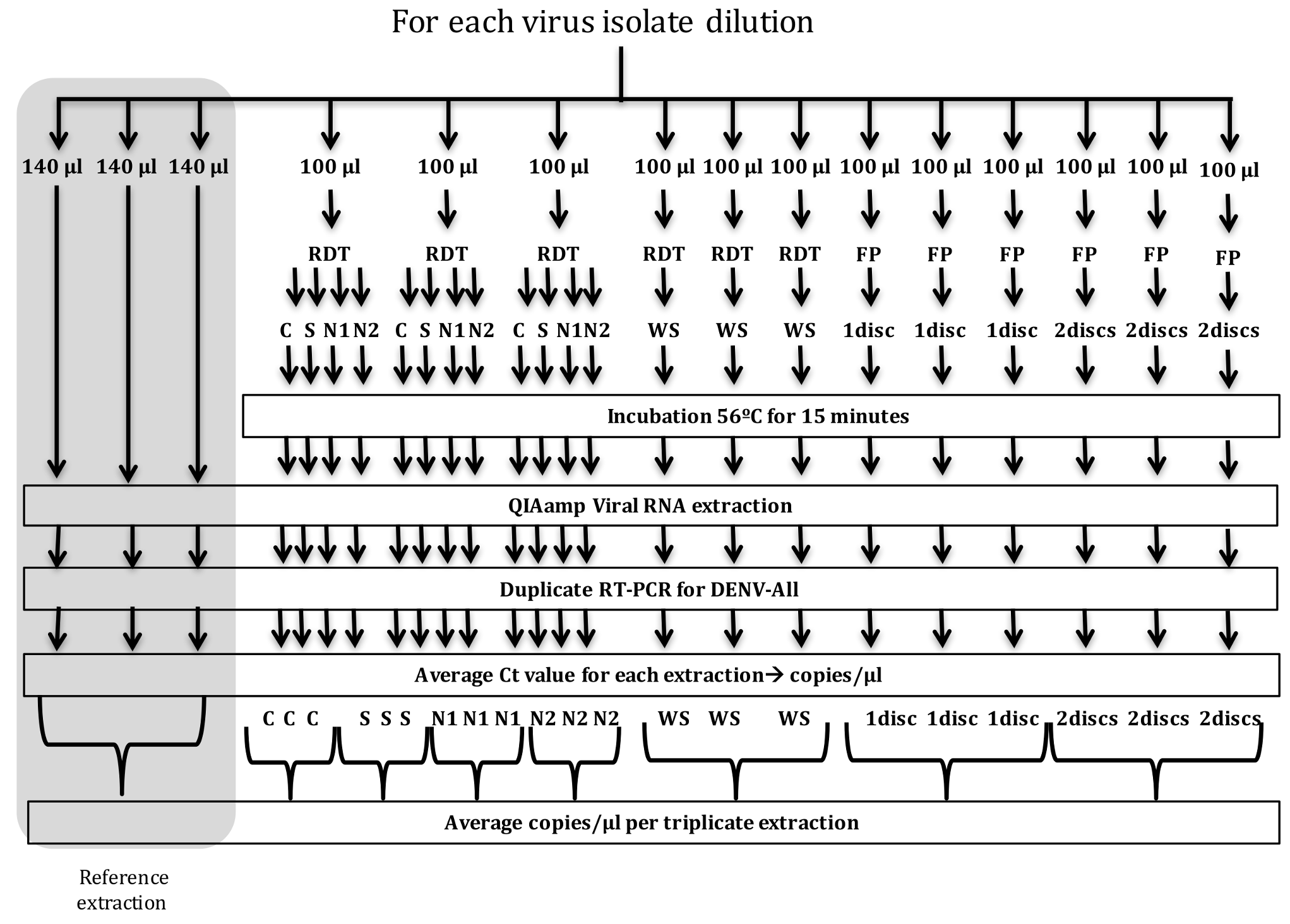

Supplement: S1 Fig — (TIF) [file pntd.0004704.s001.tif]
