# Supplementary material for: Using Rapid Diagnostic Tests as a Source of Viral RNA for Dengue Serotyping by RT-PCR - A Novel Epidemiological Tool
Source: PLoS Negl Trop Dis. 2016 May 9;10(5):e0004704. doi: 10.1371/journal.pntd.0004704 (PMC4861341; doi:10.1371/journal.pntd.0004704)
Supplement: S1 Table — (DOCX) [file pntd.0004704.s002.docx]

S2 Table. Clinical feature and laboratory RDT results for patients included in the study.

|  | Mahosot | Salavan |
| --- | --- | --- |
|  | n (%) | n (%) |
| Patient | 99 | 362 |
| Male, | 44 (44.4) | 189 (52.2) |
| Age (year), m (IQR) | 25 (20-33) | 17 (10-27) |
| Fever (≥38°C), n (%) | 59 (59.6) | 327 (91.6) |
| Days of illness, m (IQR) | 5 (4-5) | 3.5 (3-5) |
| Rash | 8 (8.1) | 55 (15.5) |
| Vomiting | 66 (66.7) | 55 (15.7) |
| Arthralgia | 23 (23.2) | 330 (93.2) |
| Myalgia | 88 (88.9) | 330 (93.2) |
| Bleeding | 38 (38.4) | 6 (1.7) |
| Fluid accumulation | 4 (4.0) | 16 (4.5) |
| Dyspnoea | 2 (2.0) | 8 (2.2) |
| Confusion | 0 | 1 (0.3) |
| Drowsiness | 0 | 5 (1.4) |
| Seizure | 0 | 2 (0.6) |
| Total white blood cells^98^, m (IQR) | 2.9 (2.3-4.9) | 5.0 (3.0-7.7) |
| Leucopenia | 72 (72.7) | 48 (47.5) |
| Haematocrit^98^ (%), m (IQR) | 40 (36-43) | 38 (34-41) |
| Platelets (10^3^ /μl), m (IQR) | 88 (53-153) | 193 (118-266) |
| Discharged alive | 97 (100) | 35 (35.0) |
| Thrombocytopenia | 73 (73.7) | 108 (30.8) |
| DF warning sign | 85 (85.9) | 102 (28.2) |
| RDT result using serum |  |  |
| NS1 RDT positive alone | 45 (45.5) | 9 (2.5) |
| IgM + NS1 RDT positive | 17 (17.2) | 6 (1.7) |
| IgM RDT positive alone | 5 (5.1) | 113 (31.2) |
| Dengue serotype 1 | 2 (2.0) | 17 (4.7) |
| Dengue serotype 2 | 5 (5.1) | 6 (1.7) |
| Dengue serotype 3 | 42 (42.4) | 0 |
| Dengue serotype 4 | 2 (2.0) | 6 (1.7) |
| Untyped | 1 (1.0) |  |

m:median, IQR: Interquartile range. p value calculated using Kruskal-Wallis test for continuous variable. * p value using Fisher’s exact test. Leucopenia: less than 4500 white blood cells per μl. Thrombocytopenia: less than 150x10^3^ platelets per μl, For patient with missing values, the superscript numbers indicate the number of patient with data.
