# Supplementary material for: Using Rapid Diagnostic Tests as a Source of Viral RNA for Dengue Serotyping by RT-PCR - A Novel Epidemiological Tool
Source: PLoS Negl Trop Dis. 2016 May 9;10(5):e0004704. doi: 10.1371/journal.pntd.0004704 (PMC4861341; doi:10.1371/journal.pntd.0004704)
Supplement: S2 Table — Dir Ext: Direct extraction, FP: filter paper. Virus isolate dilution 104 = dilutions with a virus titer of 4.3x10^4^ copies/ml, 105 = 4.3x10^5^ copies/ml and 106 = 4.3x10^6^ copies/ml. (DOCX) [file pntd.0004704.s003.docx]

S2 Table: Quantification by DENV All RT-PCR of dengue RNA recovered by FP, RDT and direct extractions from virus isolates.

|  | Dengue RNA copies/µl recovered after extraction (quantification by DENV All RT-PCR) | | | | | | | | | | | |
| --- | --- | --- | --- | --- | --- | --- | --- | --- | --- | --- | --- | --- |
|  | DENV1 | | | DENV2 | | | DENV3 | | | DENV4 | | |
| Virus isolate dilutions | 10^4^ | 10^5^ | 10^6^ | 10^4^ | 10^5^ | 10^6^ | 10^4^ | 10^5^ | 10^6^ | 10^4^ | 10^5^ | 10^6^ |
| Dir Ext1 | 4.6E+02 | 4.9E+03 | 5.3E+04 | 2.3E+02 | 1.9E+03 | 1.5E+04 | 5.4E+02 | 5.6E+03 | 4.9E+04 | 1.9E+02 | 2.2E+03 | 2.2E+04 |
| Dir Ext2 | 4.6E+02 | 4.4E+03 | 5.8E+04 | 2.7E+02 | 3.2E+03 | 1.8E+04 | 6.8E+02 | 4.6E+03 | 4.0E+04 | 2.8E+02 | 2.0E+03 | 1.4E+04 |
| Dir Ext3 | 4.3E+02 | 4.2E+03 | 6.3E+04 | 4.3E+02 | 4.1E+03 | 1.6E+04 | 5.6E+02 | 5.1E+03 | 5.6E+04 | 3.7E+02 | 2.7E+03 | 1.3E+04 |
| RDT S Ext1 | 2.6E+01 | 1.7E+03 | 8.1E+03 | 4.8E+01 | 1.2E+03 | 1.4E+03 | 1.5E+01 | 9.8E+02 | 5.6E+03 | 7.1E+01 | 8.0E+02 | 2.2E+03 |
| RDT S Ext2 | 3.3E+01 | 6.5E+02 | 6.6E+03 | 1.3E+02 | 1.5E+03 | 1.2E+04 | 6.0E+01 | 4.2E+02 | 5.3E+03 | 2.5E+01 | 4.7E+02 | 2.8E+03 |
| RDT S Ext3 | 6.5E+01 | 8.0E+02 | 8.0E+03 | 1.6E+02 | 1.8E+03 | 7.4E+03 | 4.3E+01 | 3.0E+02 | 5.5E+03 | 1.2E+01 | 3.6E+02 | 2.8E+03 |
| RDT C Ext1 | 1.8E+02 | 2.8E+02 | 1.7E+03 | 3.1E+01 | 1.2E+02 | 1.8E+03 | 1.7E+01 | 1.5E+02 | 1.3E+04 | 6.8E+00 | 1.2E+02 | 1.4E+03 |
| RDT C Ext2 | 1.4E+01 | 1.1E+02 | 8.7E+02 | 5.6E+00 | 5.8E+01 | 1.0E+03 | 1.0E+01 | 1.8E+02 | 2.0E+03 | 8.3E-01 | 4.6E+01 | 2.3E+02 |
| RDT C Ext3 | 1.7E+01 | 1.2E+02 | 1.0E+03 | 5.0E+00 | 5.9E+01 | 9.8E+02 | 1.0E+01 | 1.4E+02 | 1.6E+03 | 7.8E-01 | 3.0E+01 | 1.9E+02 |
| RDT N1 Ext1 | 2.0E+01 | 3.6E+02 | 1.2E+04 | 3.2E+01 | 1.8E+02 | 4.1E+03 | 1.2E+01 | 1.1E+02 | 1.5E+04 | 5.0E+00 | 4.4E+01 | 4.9E+02 |
| RDT N1 Ext2 | 1.8E+01 | 0 | 2.8E+03 | 1.0E+01 | 8.3E+01 | 8.5E+02 | 6.3E+00 | 7.4E+01 | 1.0E+03 | 3.5E+00 | 4.8E+01 | 3.2E+02 |
| RDT N1 Ext3 | 2.6E+01 | 2.8E+02 | 3.2E+03 | 6.2E+00 | 6.6E+01 | 9.8E+02 | 6.3E+00 | 1.0E+02 | 8.1E+02 | 3.8E+00 | 2.6E+01 | 2.5E+02 |
| RDT N2 Ext1 | 1.7E+01 | 1.7E+02 | 1.9E+03 | 6.8E+01 | 1.5E+02 | 5.3E+03 | 5.3E+01 | 1.4E+02 | 7.3E+03 | 4.6E+00 | 2.5E+01 | 2.8E+02 |
| RDT N2 Ext2 | 6.80E+00 | 8.50E+01 | 1.10E+03 | 0 | 6.00E+01 | 5.60E+02 | 5.40E+00 | 1.00E+02 | 9.40E+02 | 2.40E+00 | 3.60E+01 | 3.40E+02 |
| RDT N2 Ext3 | 8.50E+00 | 1.00E+02 | 1.00E+03 | 0 | 6.80E+01 | 8.50E+02 | 5.64E+00 | 1.00E+02 | 8.93E+02 | 4.70E+00 | 2.30E+01 | 2.70E+02 |
| RDT-WS Ext1 | 4.9E+02 | 6.6E+03 | 4.9E+04 | 3.3E+02 | 4.7E+03 | 4.1E+04 | 4.3E+02 | 1.6E+03 | 3.4E+04 | 1.2E+02 | 2.0E+03 | 2.1E+04 |
| RDT-WS Ext2 | 7.1E+02 | 7.1E+03 | 4.6E+04 | 5.5E+02 | 3.3E+03 | 3.9E+04 | 4.3E+02 | 3.2E+03 | 3.5E+04 | 2.3E+02 | 1.9E+03 | 1.6E+04 |
| RDT-WS Ext | 7.3E+01 | 6.0E+02 | 5.4E+03 | 5.0E+01 | 5.8E+02 | 3.0E+03 | 2.5E+01 | 4.2E+02 | 2.3E+03 | 3.0E+01 | 2.2E+02 | 2.0E+03 |
| FP 1 disc Ext1 | 4.2E+01 | 5.0E+02 | 4.4E+03 | 6.2E+00 | 1.5E+02 | 1.4E+03 | 5.3E+01 | 7.6E+02 | 6.9E+03 | 1.4E+01 | 7.6E+01 | 7.6E+02 |
| FP 1 disc Ext2 | 5.5E+00 | 1.5E+02 | 1.4E+03 | 0 | 2.6E+01 | 5.3E+02 | 1.2E+01 | 1.0E+02 | 1.6E+03 | 3.5E+00 | 3.0E+01 | 6.0E+02 |
| FP 1 disc Ext3 | 5.1E+00 | 1.1E+02 | 1.2E+03 | 3.6E+00 | 7.1E+01 | 4.9E+02 | 1.2E+01 | 1.6E+02 | 1.4E+03 | 2.1E+00 | 4.6E+01 | 3.8E+02 |
| FP 2 discs Ext 1 | 2.0E+01 | 1.4E+02 | 2.8E+03 | 2.9E+00 | 5.0E+01 | 5.9E+02 | 4.0E+01 | 1.7E+02 | 1.8E+03 | 6.0E+00 | 4.0E+01 | 5.1E+02 |
| FP 2 discs Ext2 | 2.2E+01 | 1.5E+02 | 2.3E+03 | 6.9E+00 | 6.8E+01 | 6.9E+02 | 4.0E+01 | 1.9E+02 | 2.6E+03 | 5.9E+00 | 4.0E+01 | 4.0E+02 |
| FP 2 discs Ext3 | 2.2E+01 | 1.9E+02 | 3.0E+03 | 4.2E+00 | 6.9E+01 | 8.7E+02 | 2.1E+01 | 3.0E+02 | 2.5E+03 | 2.8E+00 | 4.9E+01 | 3.2E+02 |

Dir Ext: Direct extraction, FP: filter paper. Virus isolate dilution 10^4^ = dilutions with a virus titer of 4.3x10^4^ copies/ml, 10^5^= 4.3x10^5^ copies/ml and 10^6^=4.3x10^6^ copies/ml.
