# Supplementary material for: Using Rapid Diagnostic Tests as a Source of Viral RNA for Dengue Serotyping by RT-PCR - A Novel Epidemiological Tool
Source: PLoS Negl Trop Dis. 2016 May 9;10(5):e0004704. doi: 10.1371/journal.pntd.0004704 (PMC4861341; doi:10.1371/journal.pntd.0004704)
Supplement: S4 Table — A: For the 99 patients admitted at Mahosot Hospital. B: For the 362 patients admitted at Salavan Hospital. (DOCX) [file pntd.0004704.s005.docx]

|  | Neat serum | RDT serum | RDT WB | FP serum | FP WB |
| --- | --- | --- | --- | --- | --- |
| Dengue 1 | 1 | 2 | 1 | 1 | 1 |
| Dengue 2 | 5 | 5 | 5 | 5 | 5 |
| Dengue 3 | 40 | 33 | 28 | 27 | 27 |
| Dengue 4 | 2 | 2 | 2 | 1 | 1 |
| Untyped |  |  | 1 |  | 1 |
| Total | 48 | 42 | 37 | 34 | 35 |

**Table S4. Results of serotyping RT-PCR performed for each RNA purification.**

A: For the 99 patients admitted at Mahosot Hospital.

B: For the 362 patients admitted at Salavan Hospital.

|  | Neat serum | RDT serum | RDT WB | FP WB |
| --- | --- | --- | --- | --- |
| Dengue 1 | 88 | 91 | 95 | 101 |
| Dengue 2 | 10 | 12 | 11 | 10 |
| Dengue 3 | 1 | 4 | 4 | 1 |
| Dengue 4 | 0 | 0 | 0 | 0 |
| Untyped | 1 | 3 | 5 | 1 |
| Total | 100 | 110 | 115 | 113 |
